# Wavelength-dependent correlation of LIPSS periodicity and laser penetration depth in stainless steel

**DOI:** 10.3762/bjnano.16.95

**Published:** 2025-08-11

**Authors:** Nitin Chaudhary, Chavan Akash Naik, Shilpa Mangalassery, Jai Prakash Gautam, Sri Ram Gopal Naraharisetty

**Affiliations:** 1 School of Physics, University of Hyderabad, Gachibowli, Hyderabad, 500046, India; 2 School of Engineering Sciences and Technology, University of Hyderabad, 500046, India

**Keywords:** cross section of LIPSS, high spatial frequency LIPSS (HSFL), laser-induced periodic surface structures (LIPSS), low spatial frequency LIPSS (LSFL), maximum LIPSS, penetration depth

## Abstract

This research paper delves into the exploration of laser-induced periodic surface structures (LIPSS) on a 100 µm thin stainless steel (SS) sheet. Through the application of laser irradiation with wavelengths spanning from 400 to 2400 nm, we systematically generate ladder-like LIPSS across a substantial area, incorporating LIPSS with both low spatial frequency (LSFL) and high spatial frequency (HSFL) simultaneously. Notably, the embedded LIPSS exhibit a linear relationship in the observed spatial periodicity of LSFL and HSFL with wavelengths up to 2000 nm, after which a decrease in periodicity is observed. By employing cross-sectional electron microscopy, we scrutinize the penetration depth of laser radiation or laser-affected zone, in the LIPSS-formed SS sheets, revealing a parallel trend with LSFL and HSFL spatial periodicity. Specifically, the penetration depth increases with wavelength up to 2000 nm, reaching a peak at approximately 13 µm, and subsequently decreases. This distinctive correlation underscores the role of plasma material reorganizational effects in LIPSS formation at higher wavelengths, presenting a new experimental observation to the existing literature. The findings enhance our comprehension of laser–material interactions and hold potential implications for surface engineering and material science applications.

## Introduction

Nanostructuring on surfaces plays a pivotal role in governing surface properties, and laser-induced periodic surface structures (LIPSS) have emerged as a potent method for achieving nanoscale surface modifications. Over the past decade, LIPSS and laser ablation techniques for micro/nanostructuring have garnered significant attention due to their versatile applications. In particular, fabricating subwavelength structures using high-power pulsed lasers offers a flexible, single-step processing approach compatible with industry standards, making it a promising alternative to high-precision lithography techniques [1–3].

The utilization of short femtosecond laser pulses has proven instrumental in overcoming diffraction limit restrictions, enabling controlled fabrication of periodic subwavelength structures [4–9]. This controlled structuring offers a straightforward means to manipulate the functional and surface characteristics of substrates [10–13]. The applications span a broad spectrum, encompassing colorization control, self-cleaning surfaces, regulation of cell and bacterial films, antireflective surfaces, surface-enhanced Raman spectroscopy, reduction of friction and wear, fuel injection, and enhancement of tribological properties [14–30].

Extensive research efforts have been directed toward understanding LIPSS, encompassing systematic investigations on different metals, semiconductors, and polymers [9,12,31–39]. LIPSS characterized by ripple-like subwavelength periodic structures on a material’s surface, are broadly classified into low spatial frequency LIPSS (LSFL) and high spatial frequency LIPSS (HSFL), based on their spatial periodicity (Λ) relative to the laser wavelength (λ). Typically in metal surfaces with high absorbance, the range of LSFL periodicity is λ > Λ > λ/2, oriented perpendicular to the incident polarization, and HSFL periodicities are much smaller and in the range of Λ < λ/2, orientated parallel to the incident polarization [40–45]. A recent review by Jörn Bonse and Stephan Gräf provides a comprehensive classification based on materials and associated theories [46].

Two primary classes of theories – electromagnetic and matter reorganization – have been proposed to explain LIPSS formation [46–48]. LSFL formation is often attributed to the interference between incoming electromagnetic radiation and surface electromagnetic waves and involves surface polaritons and surface plasmon polaritons (SPPs). These SPPs propagate along the interface of the two media in which the electron density coherently oscillates, coupled to both media. Usually, LIPSS formation is a multipulse phenomenon, as pulse after pulse create a different roughness on the surface or feedback mechanism to form certain SPP modes. The interference of incoming light with scattering from the SPP modes can lead to the modulation of the net localized energy distribution on the surface, and this field absorption is manifested as LIPSS on the surface. This process depends on several experimental factors, namely, incident wavelength, polarization, material dielectric, dielectric, fluence of the laser, pulse width, repetition rate, and number of pulses. These parameters result in different periodicities and aspect ratios, and corresponding theoretical predictions are not yet completely mature or framed. The formation of HSFL, however, remains an unrevealed phenomenon with theories ranging from twining [49] and self-organization [33] to second harmonic generation [11] and cavitation instability [23,50–51]. Despite numerous studies, the formation mechanism of HSFL and the reason for their shorter periodicity compared to the laser wavelength remain elusive. Our previous work addressed this gap by patterning a single line structure with laser irradiation wavelengths ranging from 400 to 2200 nm. From this, a linear trend in LIPSS characteristics was observed up to 2000 nm, and the threshold of the LIPSS formation was determined [52]. In the present work, we extend the LIPSS over a large area, instead of a single line, using 400 to 2400 nm laser irradiation. This enabled the examination of the cross-sectional zone where the cumulative effect of the laser irradiation occurs. We analyzed how these effects correlate with the incident laser wavelength.

The manuscript delves into the critical parameter of penetration depth, or laser-affected zone, and its impact on material processing efficiency using femtosecond laser pulses. The study reveals deviations from the expected behavior predicted by existing theories, showing a wavelength-dependent penetration depth on stainless steel. This finding challenges our understanding of how metal surfaces respond to incident wavelengths [53–54]. This work advances our comprehension of LIPSS structures and their applications, shedding new light on the interplay between incident wavelengths and surface interactions. The paper concludes by emphasizing the importance of optical properties, laser parameters, and material characteristics in determining penetration depth, thereby contributing to the broader understanding of light–material interactions.

## Materials and Methods

### Materials

A double-sided polished stainless steel surface (SS304) with a thickness of 100 µm was utilized. Before and after the laser treatment, these SS surfaces underwent ultrasonic rinsing with acetone for 10 min each, aiming to eliminate dust particles and other contaminants.

### Experimental details

**Figure 1:**
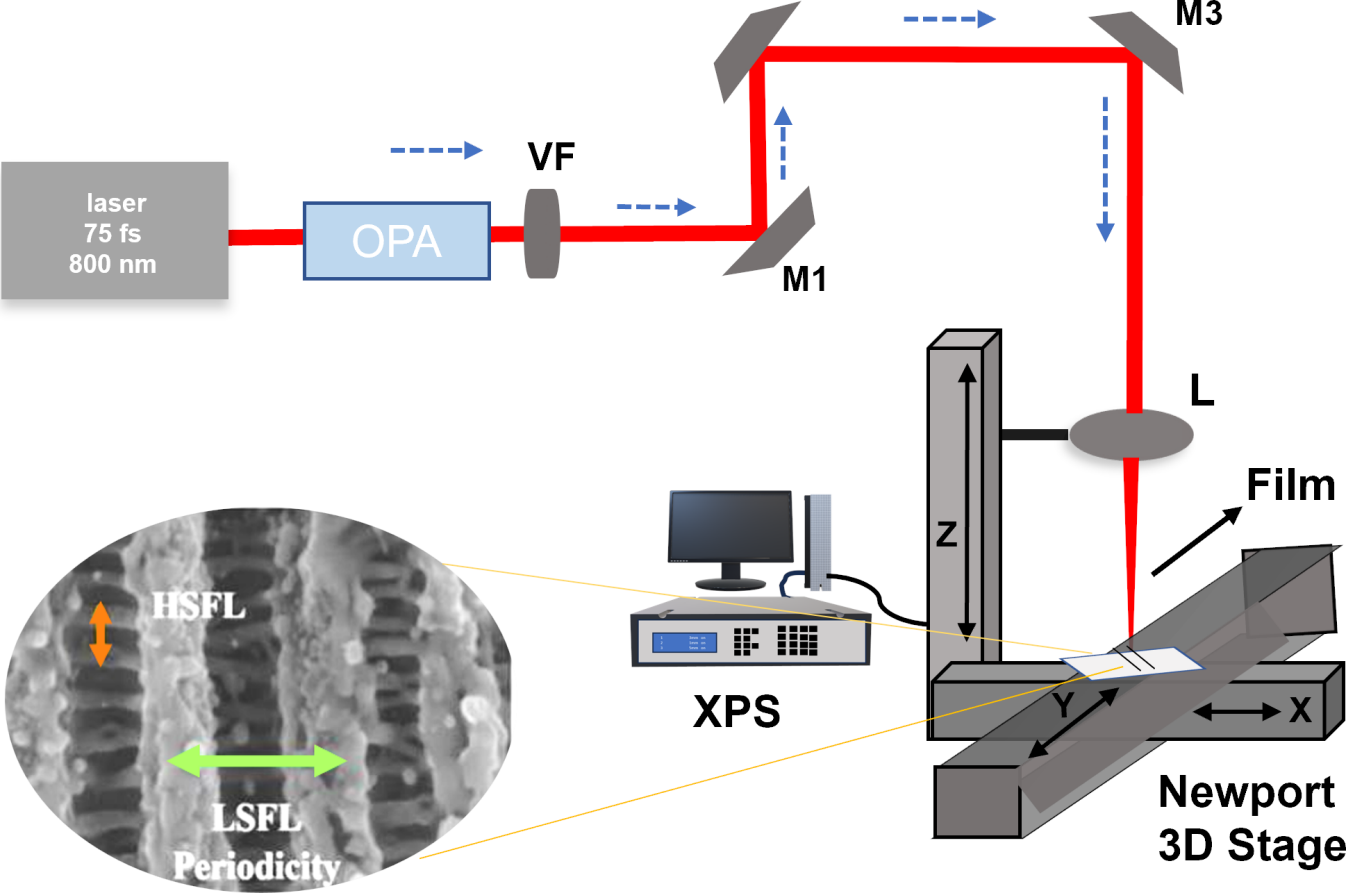
Depicts the experimental setup utilized in the fabrication process of laser-induced periodic surface structures (LIPSS). Critical components of this setup include the femtosecond laser, optical parametric amplifier (OPA), ND variable filter (VF), mirrors (M), three-axis stages (3D stages), and a convex lens (L) employed to focus the laser beam onto the surface precisely. The inset image shows the ladder-like LIPSS structures on the laser-patterned SS surface.

A commercially available Ti:sapphire femtosecond laser operating at an 800 nm central wavelength, with 75 fs pulse duration, 5.5 mJ/pulse energy, and a repetition rate of 1 kHz, was utilized for laser direct writing experiments. The fundamental part of the laser, providing 2.6 mJ/pulse, acted as the pump for an optical parametric amplifier (OPA), allowing for flexible tuning of the laser wavelength from 400 to 2400 nm. The TOPAS Prime optical parametric amplifier supplied by Light Conversion is used for this purpose. Subsequently, the laser beam was directed to ablate stainless steel following wavelength adjustments. To regulate the laser beam’s final intensity at the sample, an ND variable filter (VF) was employed as shown in Figure 1. For nanostructuring, a convex lens made of CaF_2_ with a focal length of 5 cm was used to focus the laser beam. Precise sample positioning was achieved using a three-dimensional Newport stage with a resolution of 1 µm, controlled by an ESP motion controller, with all axes being computer-controlled.

In our previous work, we had generated LIPSS on stainless steel via single-line scanning [52]. In the present work to optimize the fabrication of large-area embedded LIPSS, a series of specimens were generated using four discrete laser scanning intervals, that is, 60, 50, 40, 30, and 20 µm. It involved a meticulous process to fine-tune laser parameters such as power, speed, and step size to attain the desired LIPSS pattern. Each incident wavelength required creating samples with varying scanning speeds, specifically, 0.2, 0.5, 0.8, and 1.5 mm/s, with the input laser beam power fixed at 20 mW. For each wavelength, four different step sizes and scanning speeds were explored. Table 1 below displays the optimal spatial periodicity of LIPSS structures obtained for each wavelength. In this work, we fabricated LIPSS over a large area 5 mm × 5 mm and optimization was carried out for best ladder-like structures over a large area. The size of the sample that one can make is limited by the scanning range of the three axis stages.

### Electrolytic etching

The cross-sectional area of the laser-treated stainless steel samples underwent thorough polishing across their thickness using various grades of emery paper (3000, 4000, and 5000). Subsequently, alumina polishing was applied for 20 min to achieve a flawless mirror finish on the surface. A solution containing 10 g of oxalic acid in 100 mL of distilled water was employed for etching the stainless steel.

An external etching process was conducted to examine the surface morphology of the samples and precisely measure the depth to which the laser heat affected or penetrated the zone. Imaging was performed using an FEI NOVA NANO SEM 450 scanning electron microscope. External etching was executed using a DC power supply machine, applying a voltage of 10 kV. In this procedure, a steel plate served as the cathode and was submerged in the oxalic acid solution, while the steel sample acted as the anode and was also immersed in the solution. Etching was carried out for 80 s to unveil the microstructure.

### Characterization and measurements

High-resolution images of cross section and surface morphologies of the sample were obtained using a FESEM (Zeiss, Ultra 55). EDS was used to determine the elemental distribution on the bare and laser-treated surfaces. The periodicity in various locations on the FESEM images was determined using ImageJ software.

## Results and Discussion

### Introduction to embedded LIPSS to form ladder-like structures

The laser-induced ripples formed on surfaces typically exhibit two types of spatial periodicity, namely, LSFL and HSFL, which are generally produced at different laser fluence regimes. In most cases, the generation of LIPSS occurs at repetition rates of 100 kHz or less, where the sequence of successive pulses creates a feedback mechanism conducive to LIPSS formation. This results in pulse intervals of more than 10 µs, deemed sufficient for efficient heat conduction [55–56]. To achieve a clear formation of HSFL or LSFL, it is essential to optimize the laser direct writing (LDW) parameters, such as fluence, focusing, scanning speed, polarization, and repetition rate of the pulses. By tuning the LDW parameters, both LSFL and HSFL can be simultaneously formed on a metal surface [52]. In our previous work, we presented optimal laser parameters for the formation of ladder-like structures over a single line. In that study, the laser power/fluence was varying, and we used a fixed scanning speed of 0.2 mm/s. To cover larger areas, we need faster scanning speeds. In this work we used a fixed power for all the wavelengths and varied the scanning speed from 0.5 to 1.6 mm/s. The most important parameter for the formation of large-area LIPSS is the stepsize or line interval, which was optimized for each wavelength for the best ladder-like structures. This approach is more complex compared to single-line patterning as one has to attain structural uniformity across the entire surface [52].

A notable observation is that the periodicity of LIPSS increases when using laser wavelengths from 400 to 2000 nm, after which it decreases at both 2200 and 2400 nm irradiation. Naturally, as the photon energy decreases, the ability to ablate the material also decreases. The increase and decrease in the periodicity of large-area ladder-like LIPSS with changes in the wavelength of irradiation are explained in later sections.

The exact mechanism of LSFL formation and the reason for the shorter HSFL periods than the laser wavelength remains not fully understood, despite numerous studies on this topic. The most puzzling aspect of our current results is the decrease in the periodicity of LIPSS after irradiation with wavelengths beyond 2000 nm. Further experiments and theoretical studies are necessary to develop a precise model or understand the underlying mechanisms. In pursuit of this understanding, we monitored the formation of plasma and the depth of laser penetration inside the material for all wavelengths of femtosecond laser irradiations.

According to the plasmonic model, when the radiation is incident at a normal angle, the period of the resulting ripples can be determined using the equation [57–59]:


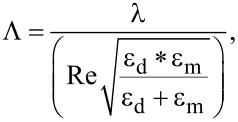


where Λ is the ripple periodicity (LSFL spatial periodicity), λ is the incident wavelength, and ε_d_ and ε_m_ are the dielectric constants of the medium and metal, respectively. ε_d_ = 1 (dielectric constant of the medium) and ε_m_ = (*n* + *ik*)^2^, *n* is the real part of the refractive index of the metal, and *k* is the coefficient of extinction. According to this model, the periodicity should exhibit an increasing trend with wavelength when there is no resonance. However, the experiments presented in this work show some anomalies.

The refractive index of stainless steel is *n* = 1.580, and the extinction coefficient is *k* = 3.413 at 500 nm [60]. The calculated ripple periodicity Λ = 470 nm is higher than most of the experimental values. This discrepancy may arise because the refractive index values of stainless steel are determined at room temperature, which might not be appropriate for metals heated with intense femtosecond laser pulses. The LIPSS period becomes smaller when the stainless steel surface is rougher as the roughness increases the real part of the refractive index at the metal–air interface [61].

### Variation of LIPSS with varying laser power

**Figure 2:**
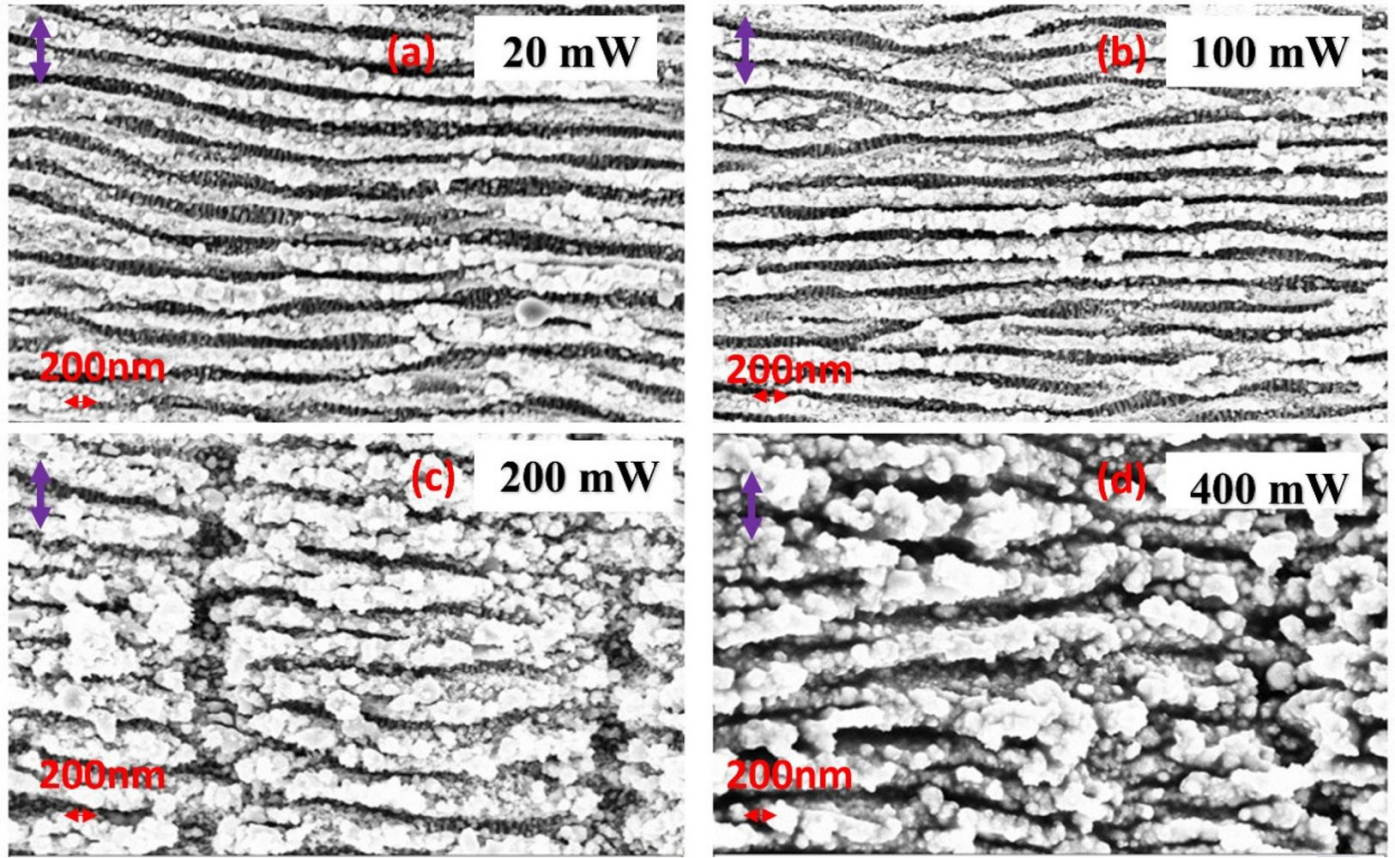
FESEM images of the surface after laser irradiation with the different incident laser powers: (a) 20 mW, (b) 100 mW, (c) 200 mW, and (d) 400 mW at 800 nm wavelength, 0.5mm/s scanning speed, and 60 µm scanning interval. The violet arrows show the polarization direction.

The spatial periodicity of LIPSS varies under different experimental conditions. This section specifically examines how surface morphology and the periodicity of LSFL and HSFL change with varying power values while maintaining a fixed wavelength of 800 nm. Figure 2 illustrates the formation of LIPSS at 800 nm for different incident powers, that is, 20, 100, 200, and 400 mW. It is observed that, at the energy levels where LIPSS form, the spatial periodicity of LSFL undergoes slight variations, while the periodicity of HSFL remains relatively constant. However, as the power values increase, HSFL begin to deteriorate, and LSFL do not manifest uniformly across the surface, as depicted in Figure 2c,d. This increased power leads to more debris accumulation, resulting in inadequate formation of HSFL. Furthermore, with an increase in power, the surface cannot sustain LIPSS altogether. We kept the scanning speed constant at 0.5 mm/s here. Attaining a stable high power at all wavelengths from 400 to 2400 nm becomes difficult with our OPAs. In the following sections, we kept a constant power output of 20 mW for all wavelengths, while the fluence was changed for each wavelength. The optimization regarding LIPSS was performed by varying the scanning speed and the step size between the consecutive lines to generate large-area LIPSS for each irradiation wavelength.

### Effect of scanning speed

**Figure 3:**
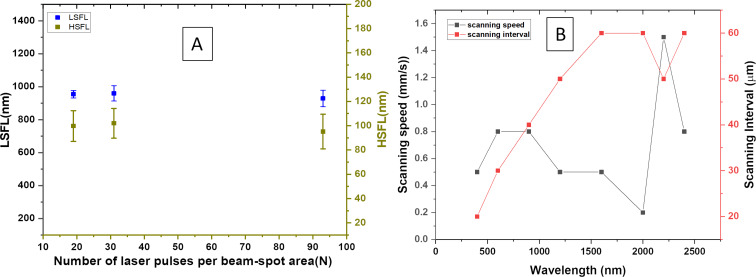
(A) Variations of spatial periodicity of LSFL and HSFL with the effective number of laser pulses per beam spot area on the incident wavelengths 2200 nm. (B) Scanning speed and scanning interval values for the best ladder-like structures with the incident wavelength.

The comparative analysis of LSFL and HSFL spatial periodicity with varying numbers of laser pulses per beam spot area is illustrated in Figure 3A for a laser wavelength of 2200 nm. When aiming to fabricate large-area LIPSS under a fixed incident laser wavelength, it becomes imperative to adjust both the scanning speed and interval carefully:


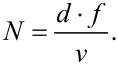


Here, *N* is number of effective laser pulses per beam spot area, *f* represents the repetition rate of the laser (1 kHz), *d* denotes the spot diameter at the focal point, and *v* signifies the scanning speed of the linear stage. As evidenced in Figure 3A, the spatial periodicity of both LSFL and HSFL remains consistent even with an increase in the number of effective laser pulses per beam spot area.

Considering that the spot diameter invariably expands with longer wavelengths, the laser-ablated area becomes more substantial in comparison to lower wavelengths. This phenomenon is crucial to note as it influences the fabrication of large-area LIPSS patterns. Perfect overlapping optimization becomes paramount in this process; any discrepancies in overlapping, whether excessive or insufficient, can lead to the destruction of LIPSS or incomplete patterning across the large surface area.

From the correlation between wavelength and spot diameter, the spot diameter of a focused laser beam increases with increasing wavelength due to the diffraction limit. Consequently, as you can see in Figure 3B, achieving optimal ladder-like structures across different wavelengths necessitates simultaneous adjustments in scanning speed and scanning interval. This coordinated approach ensures precise control over fabrication, resulting in the desired LIPSS patterns on the target material surface over a large area limited by scanning stages.

### Optimization of large-area LIPSS with the incident wavelength

As discussed in the previous section, several experimental LDW parameters influence the periodicity of the LIPSS. The scanning interval between successive lines plays an essential role in producing LIPSS on large areas of SS surfaces [33,47]. This section explains how the surface structures and spacing of LSFL and HSFL vary with exposure to wavelengths of 400, 600, 800, 1200, 1600, 2000, 2200, and 2400 nm at a constant laser power of 20 mW on a large area of stainless steel. The best ladder-like structures require optimal scanning speed and optimized step sizes between the successive laser lines, yielding clear and smooth ladder-like structures with embedded LSFL and HSFL for all the specified wavelengths over a larger area. The optimization process has been presented in detail in our previous work, where ladder-like LIPPS structures were observed over a single line [52]. In this work, we did not vary the average power of the incident beam for all wavelengths and kept it as a fixed parameter.

**Figure 4:**
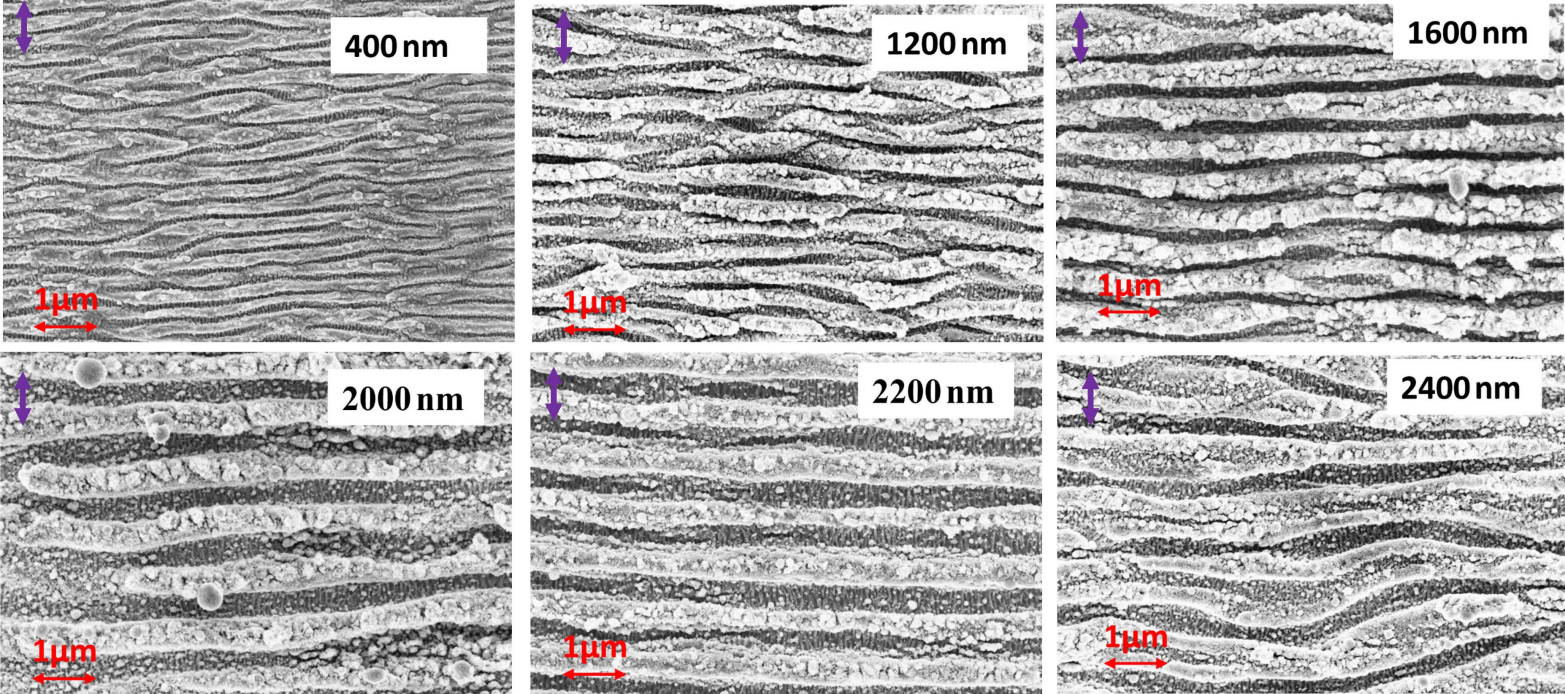
Surface patterns of the optimized ladder structures on a stainless steel surface at various incidence wavelengths.

Figure 4 presents the surface morphologies, illustrating the periodic nature of ladder-like structures resulting from exposure to varying wavelengths under fixed laser power. The focused laser spot diameter increases with the laser wavelength; hence, achieving the best ladder-like structure requires adjusting the scanning speed and step size between successive scans.

**Figure 5:**
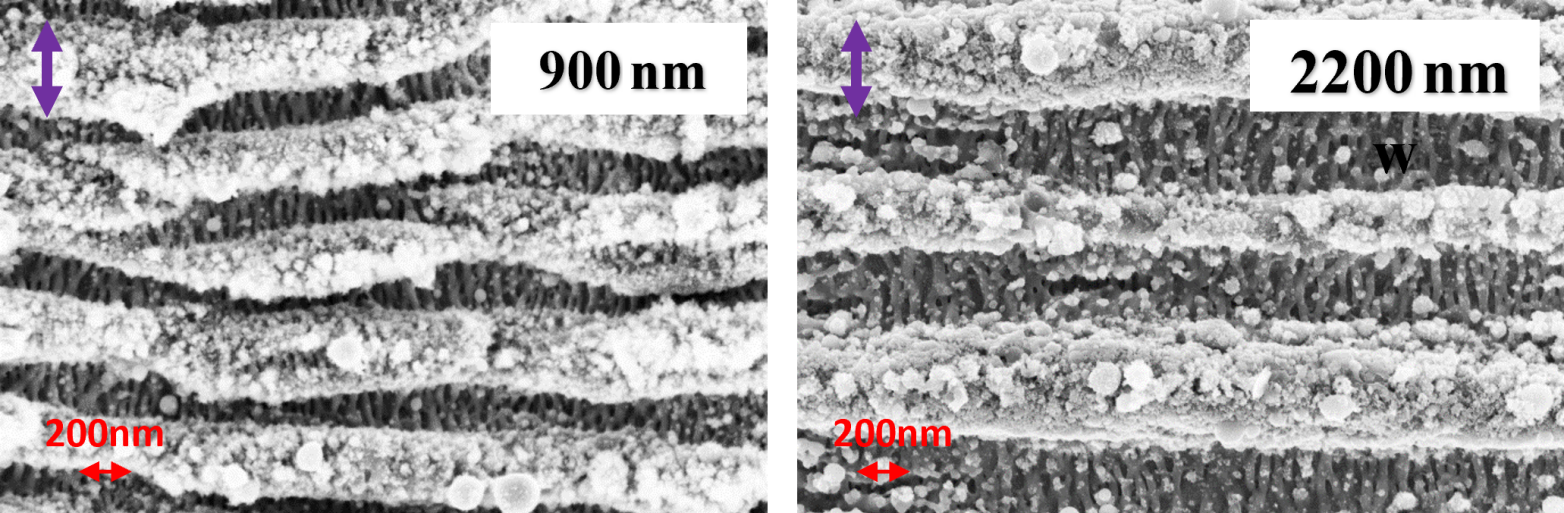
High-resolution images of ladder-like LIPSS formed at two incident wavelengths: 900 and 2200 nm. The violet arrows show the polarization direction.

Figure 4 shows the LSFL perpendicular to the incident beam polarization, with the HSFL forming deep inside the grooves of the LSFL. The orientation of these embedded HSFL is perpendicular to the LSFL and parallel to the incident beam polarization. In all FESEM images, the incident polarization direction is represented by a violet arrow at the top left corner. High-resolution images of the embedded HSFL ladder-like structure is shown in Figure 5 for two wavelengths for better understanding. The aspect ratio of these HSFL is smaller compared to the LSFL grooves and cannot be quantified accurately due to instrumental limitations. Many theories suggest that the formation of HSFL structures is due to the metal surface’s self-organization after laser irradiation [62], and some theories suggest the formation is due to the second harmonic generation at the surface [32].

**Figure 6:**
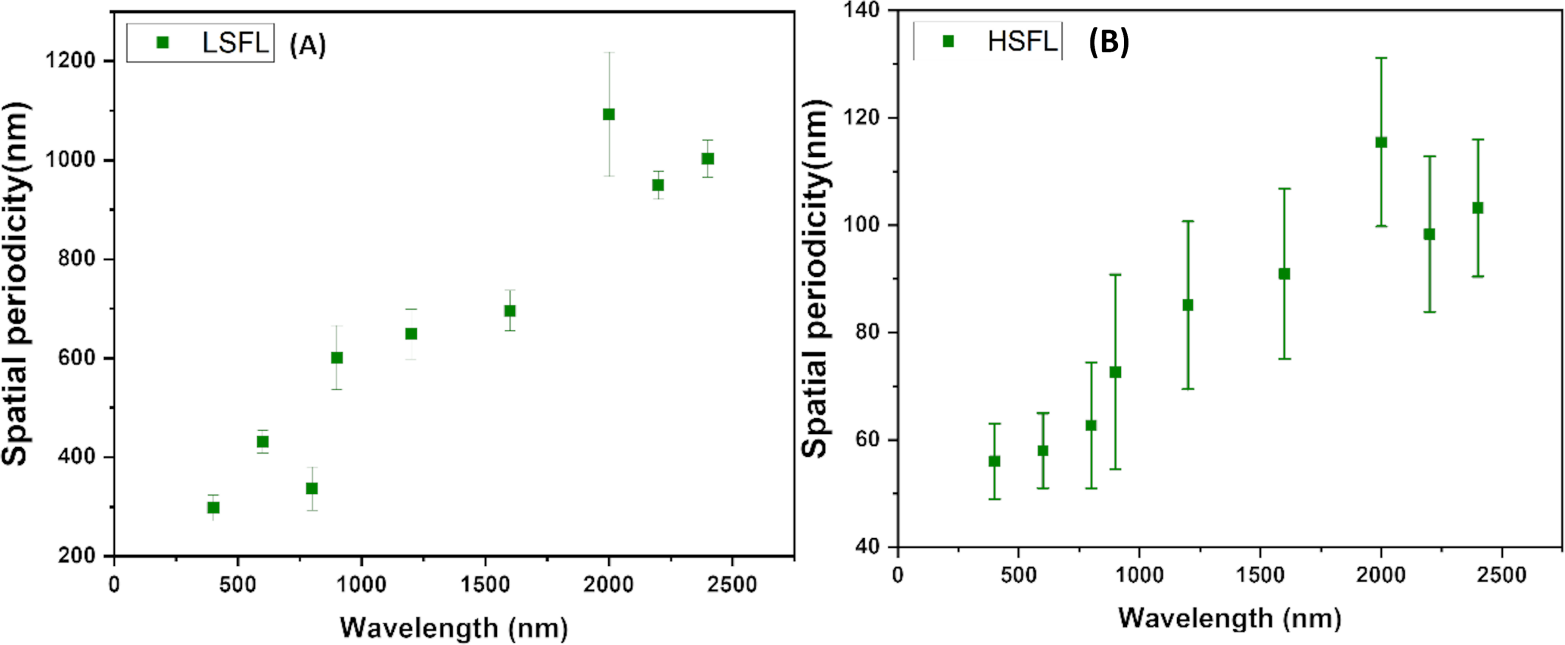
Variations of spatial periodicity of (A) LSFL and (B) HSFL with the incident laser wavelengths.

Table 1 presents the obtained LSFL and HSFL periodicities for each wavelength along with the laser irradiation parameters. The spatial periodicity of both LSFL and HSFL exhibits an upward trend as the wavelength reaches 2000 nm, but subsequently displays a decline with further increases in wavelength. This decline is less pronounced compared to our previous report, as we maintained a constant incident power. The HSFL variation trend is very similar to the LSFL trend, as evident from Figure 6, but their orientation is perpendicular to the LSFL and embedded inside the grooves.

**Table 1:** LSFL and HSFL spatial periodicities for each wavelength and corresponding parameters.

Incident wavelength (λ) nm	Spot diameter (µm)	Optimal fluence (J/cm^2^)	Effective number of pulses per spot	Λ_LSFL_ (nm)	Λ_HSFL_ (nm)

400	38	49.1	17	298 ± 26	56 ± 7
600	35.7	26.2	16	431 ± 23	58 ± 7
800	17	9.8	24	337 ± 44	63 ± 12
900	52.1	4.9	49	601 ± 65	73 ± 18
1200	50.9	2.7	51	649 ± 50	85.0 ± 15
1600	59.9	5.4	66	697 ± 40	91 ± 16
2000	84.9	2.0	212	1093 ± 126	115 ± 16
2200	93.4	0.8	93	949 ± 28	98 ± 15
2400	95.5	0.6	119	1003 ± 37	103 ± 13

### Cross-sectional depth measurements

In the previous section, we examined how LIPSS vary with the wavelength of the laser and how they behave under different laser scanning speeds, and scanning intervals. In this section, to investigate the reason behind the variation in the periodicity of LIPSS, we probe the laser penetration depth of the LIPSS structure at different wavelengths. Penetration depth could be a key parameter determining the efficiency and quality of material processing by femtosecond laser pulses. SPPs also experience energy loss due to absorption in the metal and scattering in other directions, which depend on the properties of the metal [43]. The penetration depth of SPPs into the metal measures how far the electric field of the SPP decays exponentially inside the metal.

Ash and colleagues reported the impact of wavelength and beam width on tissue penetration during light–tissue interaction. They found that higher wavelengths result in greater penetration depth, while larger spot sizes do not significantly increase penetration depth [63]. The penetration depth depends on the material’s dielectric properties and the interface’s geometry. It can also depend on the optical properties of the material, such as the refractive index, absorption coefficient, reflectivity, and laser parameters, such as the wavelength, pulse duration, fluence, incidence angle, and polarization [64]. Generally, penetration depth increases with increasing wavelength and decreases with increasing metal conductivity [53]. In the literature, most researchers used high-intensity pulse lasers for works on laser welding and substrate melting [57–58,61,63–67]. However, we could not find any experimental works dedicated to unraveling the penetration depth of LIPSS for the broadband in the existing literature.

As we know, energy penetration depth under intense femtosecond laser irradiation can be described by the following equation:


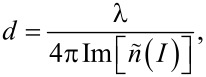


where *d* is the skin depth of the material, 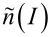 is the intensity-dependent complex refractive index, and 
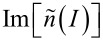
 is the imaginary part of the refractive index, responsible for absorption at the given intensity [68–69]. If the incident light wavelength increases, the skin depth of the material also increases. This means that longer wavelengths can penetrate deeper into the metal than shorter wavelengths, creating a larger plasma volume. However, under femtosecond laser processing, the material response depends on specific properties of the material, such as plasma formation, nonlinear absorption, and multiphoton ionization. In such a high-intensity regime, the optical constant of the material becomes dynamically dependent on the laser intensity. In the linear regime, pure metals such as copper, aluminum, and silver, which have low electrical resistivity, exhibit a low penetration depth for EM waves. In contrast, composite alloys such as stainless steel have higher resistivity and show a higher penetration depth than pure metals [70–72].

**Figure 7:**
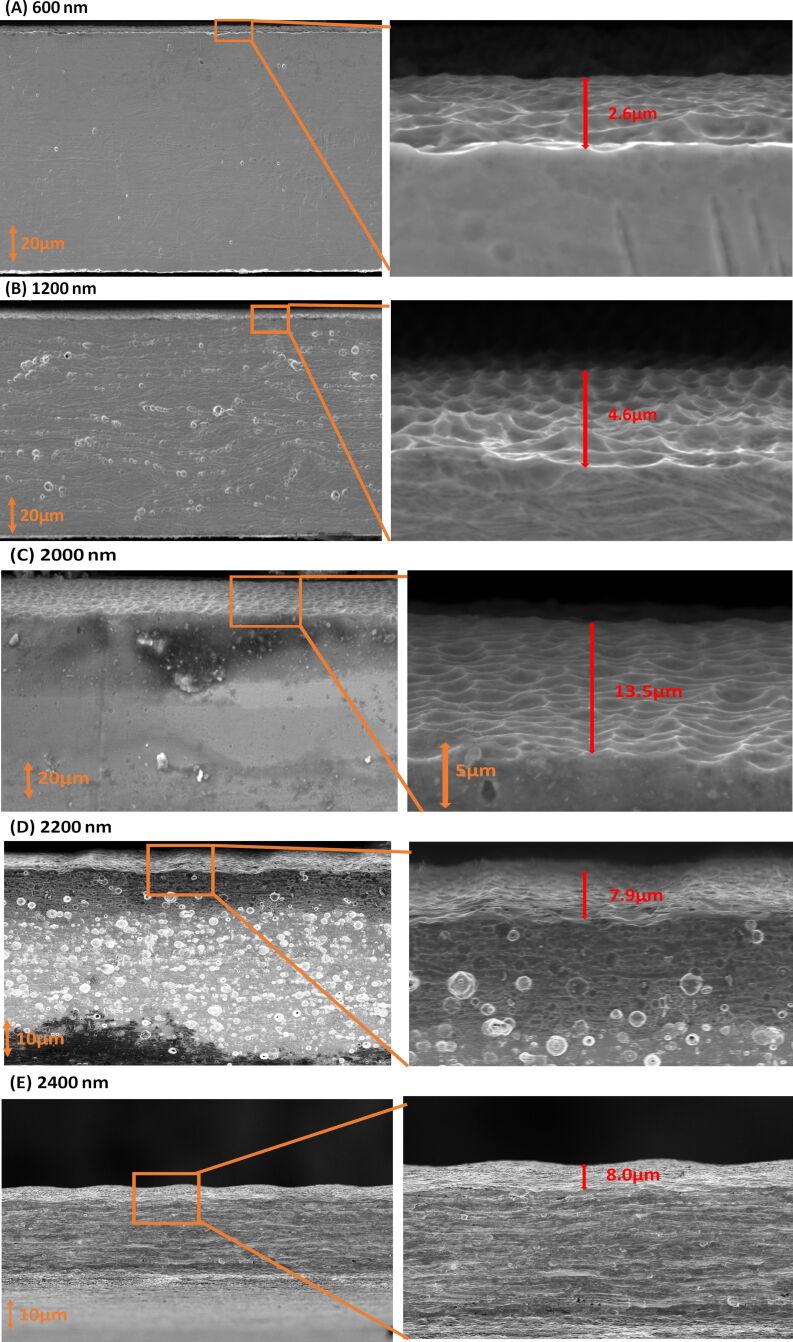
FESEM cross-sectional images of samples irridiated with wavelegths of (A) 600 nm, (B) 1200 nm, (C) 2000 nm, (D) 2200 nm, and (E) 2400 nm.

**Figure 8:**
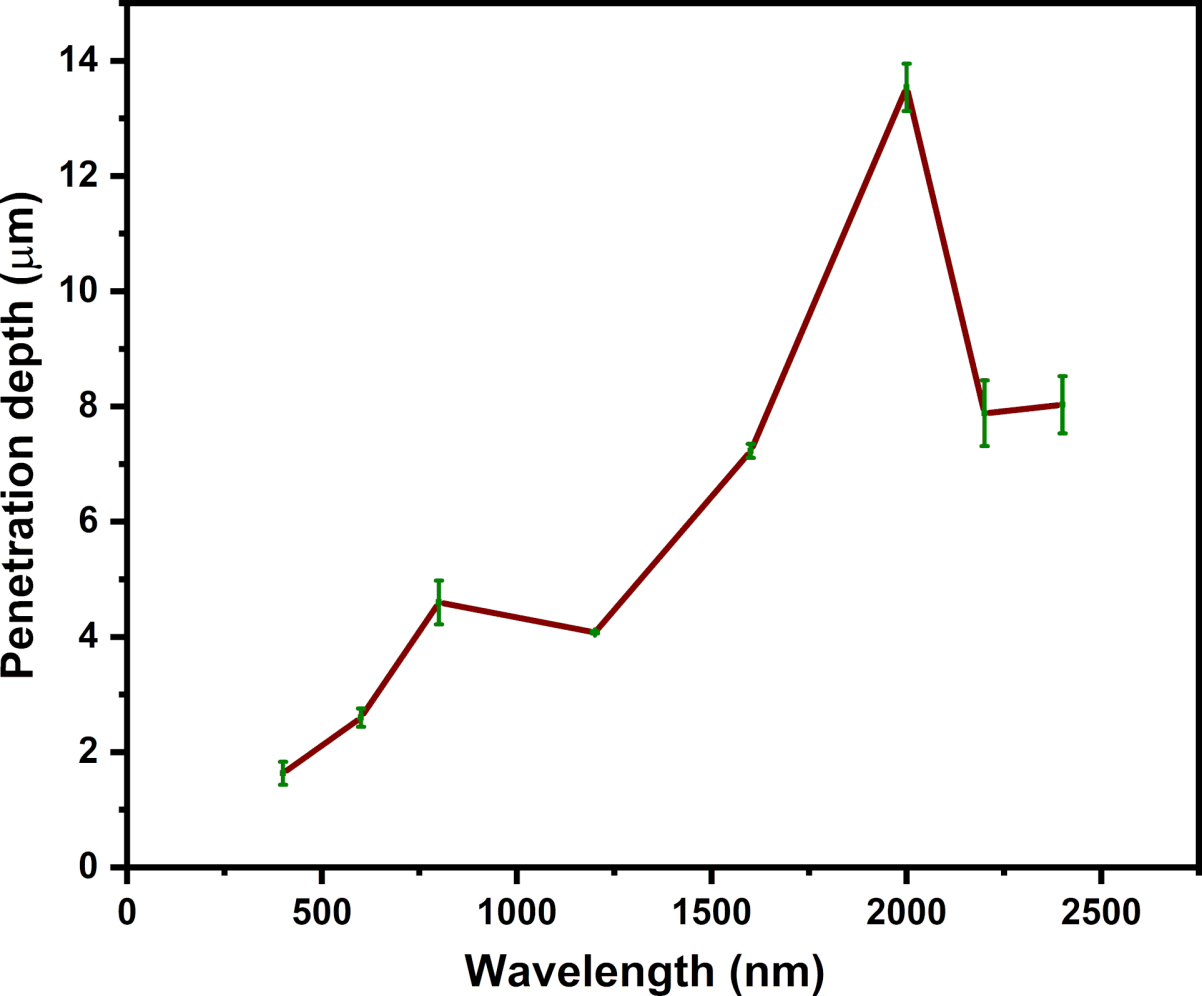
Penetration depth as function of the incident wavelength (400–2200 nm).

Figure 7 displays cross-sectional surface morphology images for samples irradiated with 600, 1200, 2000, 2200, and 2400 nm laser wavelength. As depicted in Figure 8 and Table 2, the diagram reveals a contradiction to the established principle of skin depth, as the depth of penetration increases with increasing wavelengths up to 2000 nm. However, beyond 2000 nm, the penetration depth in stainless steel diminishes. The trend of penetration depth variation is almost similar to the LSFL periodicity variation with wavelength, as shown in Figure 6A. This indicates that the amount of plasma created by the femtosecond laser on the surface plays a role in determining the periodicity of the LIPSS. In general, the plasma properties depend on the material, including the number of electrons present per unit volume in the plasma and their characteristic frequency. The frequency at which unbound electrons within stainless steel oscillate in the presence of an electromagnetic field is known as the plasma frequency. The metallic substance’s electron density and effective electron mass also affect the plasma frequency.


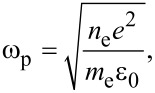


where ω_p_ is the plasmonic frequency, *n*_e_ is the electron density, and *m*_e_ is the effective mass of the electron.

**Table 2:** Shows the penetration depth and aspect ratio corresponding to the different incident laser wavelengths.

Wavelength (nm)	Penetration depth *d* (µm)

400	1.7 ± 0.1
600	2.6 ± 0.2
800	4.5 ± 0.3
1200	4.1 ± 0.0
1600	7.2 ± 0.1
2000	13.5 ± 0.4
2200	7.9 ± 0.6
2400	8.0 ± 0.5

Stainless steel has a more complex electron density than pure metals because it is composed of a mix of iron, chromium, nickel, and other elements. Stainless steel’s electron density varies depending on the composition and microstructure of the alloy, typically falling around 10^22^ to 10^23^ cm^−3^ [73–74]. It is conceivable that the plasma frequency can have an effect at wavelengths exceeding 2000 nm, leading to a reduction in penetration depth for larger wavelengths (2200 and 2400 nm); detailed theory and experimental work are needed with pure metals to confirm this.

### Depth of the patterned substrate at different powers

**Figure 9:**
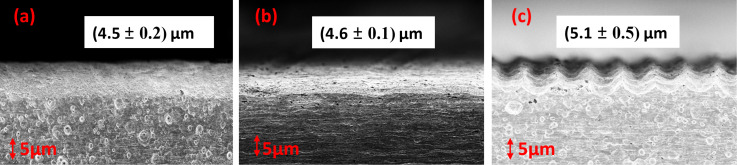
Penetration depth as function of the incident power at a wavelength of 800 nm. (a) 20 mW, (b) 150 mW, and (c) 300 mW.

In this section, we investigated the penetration depth of irradiated stainless steel samples across different laser power values, employing a single wavelength of 800 nm. As the power is increased, it is well known that more material from the surface can be removed. However, the point of investigation is how the laser-affected zone or penetration depth varies with power in the remaining part of the material. To explore this, we irradiated large areas of stainless steel surfaces using laser powers of 20, 150, and 300 mW at a constant scanning speed of 0.8 mm/s. LIPSS are observed up to 150 mW. At higher powers, these structures are destroyed. As highlighted in our previous discussions, the integrity of the LSFL and HSFL began to deteriorate at significantly high fluence values (Figure 2). Regardless of the HSFL quality of formation, we investigated the penetration depth of the laser with increasing incident power. At higher powers, the depth of laser ablation into the material increases. Delving deeper, Figure 9 offers a cross-sectional view of the large-area patterned surface, providing crucial insights into the observed phenomena.

Our analysis revealed that the average penetration depth varied marginally across different fluence levels, that is 4.5 µm for 20 mW, 4.6 µm for 150 mW, and 5.1 µm for 300 mW (Figure 9). This observation leads us to conclude that under fluences of optimal LIPSS conditions, the penetration depth exhibits minimal variance in response to fluctuations in fluence levels.

### Elemental distribution on the surface of LIPSS at different wavelengths

When subjecting a material’s surface to pulsed laser irradiation, a fascinating phenomenon occurs, namely, the ablation of material along the laser path, accompanied by redistribution of metallic particles in the surrounding areas. This rapid process leads to the formation of intricate surface nanostructures, as elucidated in the previous sections. Here, we examine whether the fundamental composition of SS undergoes any alterations with varying incident wavelengths.

To investigate this, we conducted energy-dispersive X-ray spectroscopy (EDS) analysis on laser-treated stainless steel samples across different wavelengths alongside untreated stainless steel for comparison, as outlined in Table 3. Specifically, we examined the weight percentage of Cr, Fe, and Ni in three distinct areas of the laser-treated surface exhibiting ladder-like LIPSS.

**Table 3:** Weight percentages of ablated stainless steel surface on different incident wavelength.

Elemental analysis by EDS			

Wavelength (nm)	Cr (wt %)	Fe (wt %)	Ni (wt %)

400	20.73	73.46	8.69
600	20.66	73.48	8.88
800	20.54	72.85	8.56
1200	20.33	72.06	8.74
1600	20.52	73.48	8.37
2000	20.56	73.48	8.37
2200	20.41	70.96	8.64

**Figure 10:**
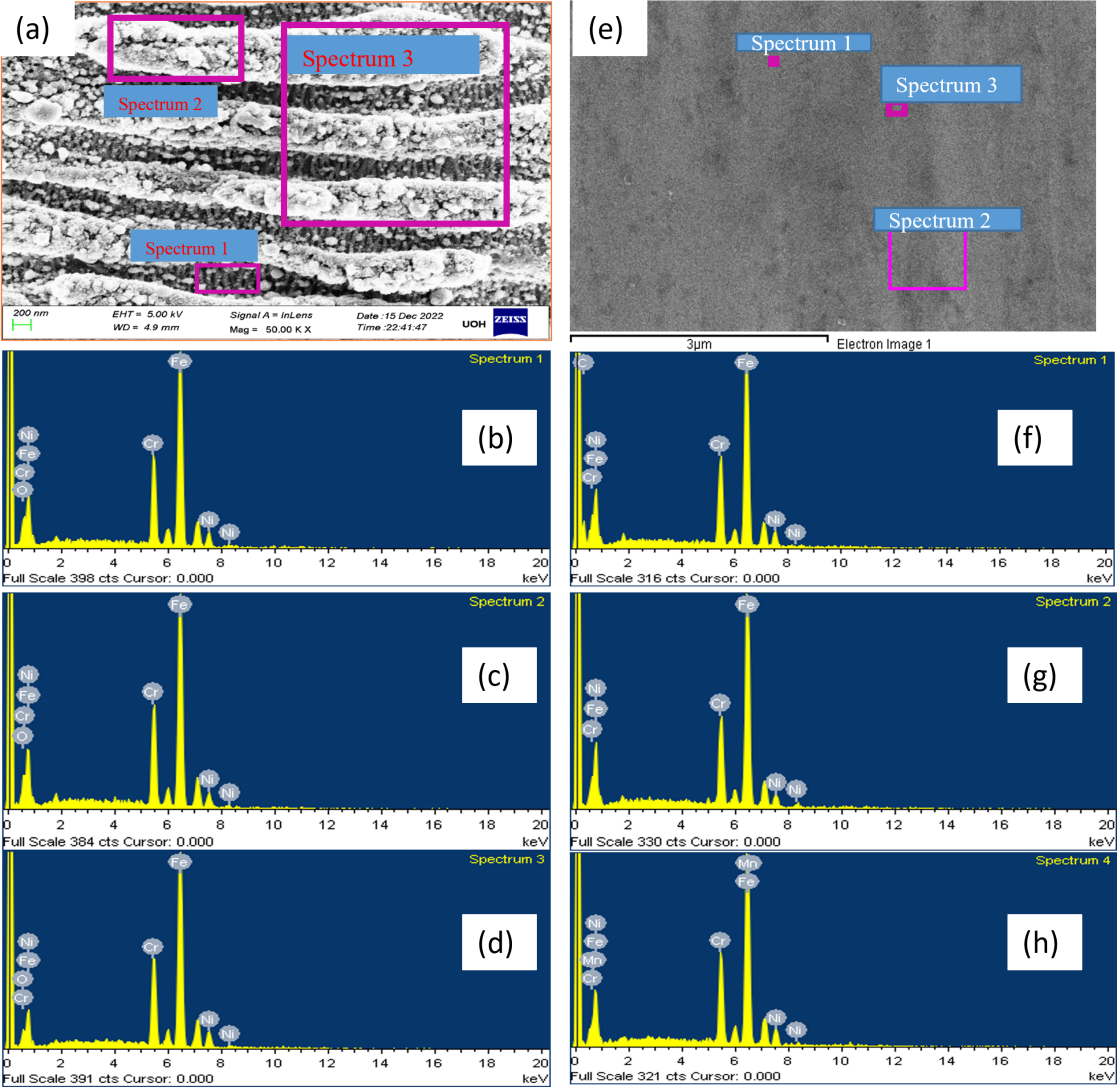
Showing the EDS elemental analysis for laser-treated and untreated stainless steel surface. (a) SEM image of laser-treated SS substrate, we picked three areas whose EDS spectra are shown in b, c, and d. (e) SEM image of bare SS surface, (f–h) elemental distribution corresponding to the different untreated polished stainless steel substrate areas.

Figure 10b–d present spectra corresponding to the three regions, that is, deep inside the grooves, at the upper side of the grooves, and at a large area (as depicted in Figure 10a). Remarkably, the EDS analysis revealed consistent weight percentages of Cr, Fe, and Ni across all three areas. This uniformity suggests that the elemental composition remains unchanged throughout the whole laser-treated surface.

For a comparative study, we also measured the elements on a polished stainless steel surface, depicted in Figure 10e. Figure 10f–h exhibits the EDS spectra of three different regions of the untreated surface, highlighting the presence of Cr, Fe, Ni, Mg, and C. However, magnesium and carbon are comparatively lower in abundance and not uniformly distributed in the unablated sample, and these peaks do not appear after the ablation at all wavelengths. This slight variation in elements between the laser-treated and untreated samples can be attributed to the highly intense femtosecond laser and its interaction with matter and the fact that these low-atomic-weight species could have escaped from the system.

## Conclusion

For the first time, we demonstrated the fabrication of ladder-like LIPSS over a large area, with controllable periodicities ranging from 250 to 1200 nm by selecting the appropriate femtosecond laser irradiation wavelength. These controlled nanoscale LIPSS can be created over a large surface area, limited only by the scanning range of the instruments, offering a facile method for industrial applications. Our findings revealed that the periodicity of LIPSS increased with the laser wavelength up to 2000 nm, followed by a decrease at 2200 and 2400 nm. Current theories and mechanisms could not explain this anomalous trend, indicating a complex interplay of factors influencing material processing by femtosecond laser pulses. We investigated cross-sectional depth measurements on fabricated sheets to unravel this puzzle. Our studies demonstrated a perfect correlation between the penetration depth of the laser at each wavelength and the periodicity achieved at that wavelength. These results reveal that the reorganization of the material or the plasma created by the femtosecond lasers play an important role in forming LIPSS, along with the electromagnetic interactions of the surface plasmon modes. Furthermore, our EDS analysis showed that the material distribution is homogeneous, regardless of the irradiation wavelength. Overall, our study provides valuable insights into the mechanisms and optimization of LIPSS formation on stainless steel surfaces using femtosecond laser pulses. The findings have significant implications for developing new theories of light–matter interaction and various applications such as surface functionalization, microfabrication, and developing advanced materials with tailored surface properties.

## Data Availability

All data that supports the findings of this study is available in the published article and/or the supporting information of this article.
